# Validity and reliability of the Turkish version of the orthognathic quality of life questionnaire in patients with dentofacial deformity

**DOI:** 10.4317/medoral.25276

**Published:** 2022-06-05

**Authors:** Duygu Turna, M- Emre Benlidayı, Ayça Üstdal Güney, Yaşar Sertdemir

**Affiliations:** 1 ORCID ID: 0000-0001-6722-4699. Department of Oral and Maxillofacial Surgery, Faculty of Dentistry, Çukurova University, Adana, Turkey; 2 ORCID ID: 0000-0002-6102-9136. Department of Oral and Maxillofacial Surgery, Faculty of Dentistry, Çukurova University, Adana, Turkey; 3 ORCID ID: 0000-0002-3190-864X. Department of Orthodontics, Faculty of Dentistry, Çukurova University, Adana, Turkey; 4 ORCID ID: 0000-0003-4455-3590. Department of Biostatistics, Faculty of Medicine, Çukurova University, Adana, Turkey

## Abstract

**Background:**

Due to the lack of a specific quality of life (QoL) survey on dentofacial deformities (DFD) for Turkish speakers, the present research aimed to perform a translation of the English version of the Orthognathic Quality of Life Questionnaire (OQLQ) into Turkish (the OQLQ-TR) and provide cultural adaptation to the Turkish population.

**Material and Methods:**

The process of this cross-cultural adaptation followed the six stages given in the guidelines that were proposed by Beaton *et al*. (2000), which comprised the following: 1) performing the initial translation, 2) synthesizing the translation, 3) performing the back translation, 4) presenting it to the expert committee, and 5) testing the prefinal version. Throughout the process of validating the Turkish version, the results of the OQLQ were compared with the Oral Health Impact Scale-14 (OHIP-14) and Short Form-36 (SF-36) questionnaires and the Visual Analogue Scale (VAS), which were previously validated in Turkish. All of these Turkish questionnaires (OHIP-14, SF-36, OQLQ) were applied to 69 patients at the Çukurova University Faculty of Dentistry.

**Results:**

Analysis of the internal consistency of the OQLQ-TR exhibited good correlations for the domains. Moreover, the test-retest reliability also exhibited intra-class correlation coefficients that were excellent. The correlation between the OQLQ-TR and SF-36 was weak and negative. The OQLQ-TR exhibited good correlations with the OHIP-14 and VAS.

**Conclusions:**

The OQLQ-TR was found to be valid, reliable, and reproducible. Thus, it has become a useful instrument for assessing the quality of life of Turkish-speaking patients with dentofacial deformity.

** Key words:**Dentofacial deformity, orthognathic quality of life, quality of life (QoL), orthognathic surgery.

## Introduction

Dentofacial deformity (DFD) is a developmental clinical situation that describes positional abnormalities of the maxillomandibular complex, resulting in malocclusion (1). Patients who have DFD may suffer from unsatisfactory chewing, phonation, and facial appearance or combination of these symptoms (2-4). All of the symptoms may affect the patient’s quality of life (QoL) (5). Currently, the most frequently used treatment for facial deformities is the combination of orthognathic surgery and orthodontic treatment (6). The impact of dentofacial deformities and their treatments on the individual’s quality of life should be evaluated and measured with appropriate instruments which should be sensitive and reliable.

The researches have only been focused on the downsides and progressions of the deformity, while in past decades, there has been increasing interest with regard to measuring both the negative and positive changes in oral health status (7,8). The need for instruments that can measure the QoL related to oral health has led to the development of various documents (9). The OQLQ is one of the most generally known patient-based questionnaires and consists of items about DFD (9-11). Therefore, it has been translated into various languages since its first publication in 2000 (10-17).

There is no specific QoL questionnaire with regard to DFDs for Turkish-speaking people. Due to this lack of knowledge, the aim of the current research was to perform a translation of the OQLQ into Turkish and examine the reliability and validity of Turkish version of the OQLQ (OQLQ-TR) on patients who have DFDs. Therefore, a clinically reliable and valid questionnaire for QoL will be presented for clinicians in Turkey during pre- and postoperative clinical evaluation. This questionnaire can also serve to contribute to QoL or evaluate the outcome of the treatment in this process.

## Material and Methods

This research comprises a cross-cultural adaptation of the OQLQ into Turkish. The OQLQ was first developed by Cunningham *et al*. in 2000 (11) and then also validated by Cunningham *et al*. in 2002 (10), respectively.

Patients with DDF who applied to the Cukurova University Faculty of Dentistry were included in the study.

Ethical approval for this study was waived by the Ethical Committee for Human Research. Additionally, informed signed consent was given by all of the participants (No: 83, December 7, 2018).

All participants who fulfilled the following criteria were accepted for participation in the study:

a) Being over 18 years of age.

b) Having Class II or class III skeletal deformity, laterognathism, vertical maxillary excess, anterior open-bite, or any combination of these conditions.

c) Not having started orthodontic treatment before the surgery.

Exclusion criteria:

a) Having previously had orthognathic surgery

b) Not having any teeth

c) Having a cleft lip and palate

d) Having a craniofacial deformity

The cultural adaptation process was carried out in accordance with the guide published by Beaton *et al*. (18). This procedure consisted of 6 stages. Stage I consisted of the initial translation. Stage II consisted of synthesizing the translation. Stage III consisted of the performing a back translation of the Turkish version. Stage IV consisted of presenting the translation to an expert committee. Stage V consisted of testing the pre-final version. Stage VI was an appraisal process in which it was suggested that an advisory committee and/or the developers conducted a review of the process and then determined if the translation was accepTable or not (18). During the verification process, the results of the OQLQ is compared with Turkish version of OHIP-14 (Oral Health Impact Profile-14), SF-36 (Short Form-36) and VAS (Visual Analog Scale) (19,20).

The OQLQ aims to assess the impact of DFDs as well as the benefits of orthodontic-surgical treatment on the QoL of the patients. This questionnaire has been widely used by researchers and comprises 22 questions that are divided into 4 domains: social aspects of deformity, facial aesthetics, oral function, and awareness of facial deformity. Patients can choose options from a 4-point scale, which vary from ‘it does not bother me’ (0 points) to ‘it bothers me a little’ (1 point), to ‘it bothers me a lot’ (4 points). OQLQ total scores can varied from 0 to 88. A lower score suggests the improvements in the QoL, whereas a higher score suggests that the QoL of the patients has become even worse (10,11).

The OHIP is a questionnaire that comprises 14 questions, which was developed in 1994 by Slade and Spencer. It was conceived in order to be able to measure the ways in which various oral conditions have an effect on QoL. In terms of subjects, OHIP is divided into 7 main dimensions, which comprise 1) functional limitations, 2) physical pain, 3) psychological discomfort, 4) physical disability, 5) psychological disability, 6) social disability, and 7) handicap. Turkish validity and reliability studies were previously performed for the OHIP-14 (the OHIP-14-TR) (19). The results were evaluated using a 5-point Likert-type scale, in which 0: None, 1: Rarely, 2: Sometimes, 3: Often, and 4: Very often

The Short Form-36 (SF-36) QoL questionnaire, which was previously validated in Turkish (20), consists of a self-rating scale with generic criteria. This questionnaire contains 36 statements that are divided into 8 dimensions, which comprise 1) physical functioning, 2) physical role difficulties, 3) pain, 4) general health, 5) vitality/energy, 6) social functioning, 7) emotional role difficulties, and 8) mental health. For each one of these dimensions, the raw data were converted and summed on a scale of 0 to 100, in which a higher score indicated a better state of health.

A sample group, which consisted of 82 patients, was selected at the Faculty of Dentistry of Çukurova University. The patients were administered the 3 questionnaires, consisting of the OQLQ-TR, the OHIP-14, and the SF-36, as well as the VAS, which ranged from 0 to 100, in which a higher score indicated a lower state of health. 10 of the patients who meet the inclusion criteria participated in a pilot study to determine the understandability of each of the questions and they were not included in the general study. The OQLQ-TR, OHIP-14, SF-36, and VAS were applied to 72 patients but the data of 69 patients were found to be valid and included in the statistics. A week after the questionnaires and the VASs had been filled-out and collected, the patients were all asked to complete the OQLQ-TR a second time (Stage 2).

- Statistical Analysis

Statistical analyses were performed using IBM SPSS Statistics for Windows 20.0 (IBM Corp., Armonk, NY, USA). The categorical variables were expressed as numbers and percentages, whereas the continuous variables were expressed as the mean and standard deviation, and the median and minimum-maximum where appropriate. For a comparison of the continuous variables between two groups, the student t test was used. The Pearson correlation coefficient and the corresponding P-value were obtained to evaluate the correlation between the continuous variables. Intraclass correlation coefficients were obtained and demonstrated. Statistical significance was accepted as *p* < 0.05 for all of the analyses.

## Results

The mean age of the 69 patients was 23.57 ± 6.61 years. Moreover, 45 of the patients (65.2%) were female and 24 (34.8%) were male. Of the patients, 6 had Class I malocclusion (anterior open bite), 23 had Class II, and 40 had Class III (Table 1).

- Reliability

The results of the internal consistency (ICC) analysis to check the reliability of the scale is given in Table 2.

**Table 1:**
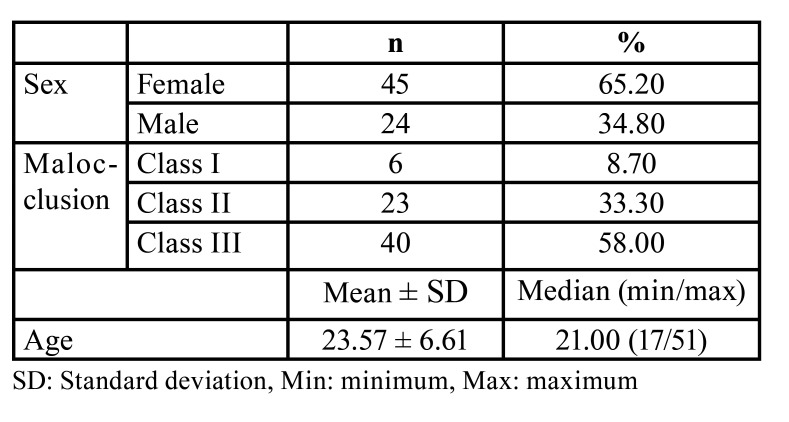
Demographic information of the participants.

**Table 2:**
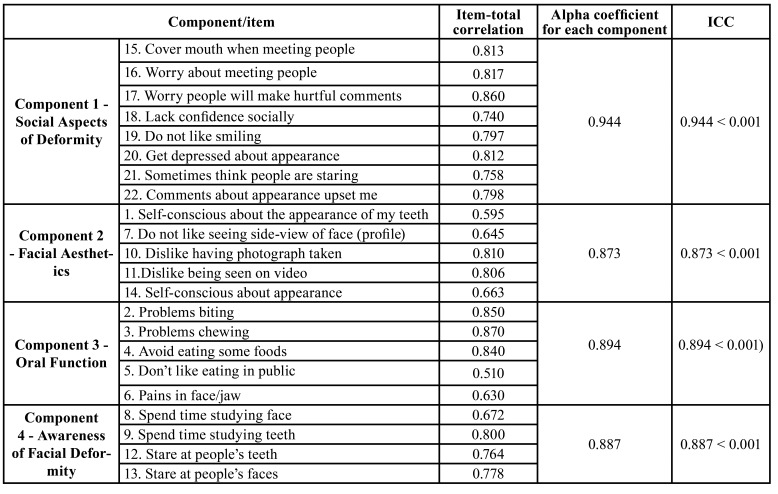
Reliability of the Turkish version of the OQLQ (n = 69).

The ICC was found to be significantly higher in all of the items and domains (*p* < 0.001) (Table 2). All of the components and domains of the OQLQ-TR showed high correlations, although the highest score was obtained in Component 1- Social Aspects of Deformity (0.944 < 0.001).

The test-retest reliability of the OQLQ-TR (OQLQ versus. OQLQ- TR2) showed excellent ICCs, and was equal or superior to 0.94 (Table 3).

- Validity

The OHIP-14 domains of social disability, psychological disability, and handicap had a higher impact on the QoL of individuals who had DDF. In these patients, a decreased level of QoL, as evaluated using the generic SF-36 form, was seen in the physical functioning, mental health, social functioning, and pain domains. The scores of all of the OQLQ domains showed significant moderate to high correlation with the VAS global self-rating. The highest correlations were for the domain of oral function.

The correlation matrix that was determined between the OQLQ domains and the SF-36, OHIP, and VAS are given in (Table 4).

**Table 3:**
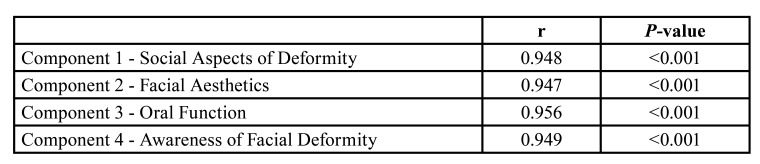
Correlations between the 1st and 2nd applications of the questionnaire (test-retest reliability of OQLQ-TR).

**Table 4:**
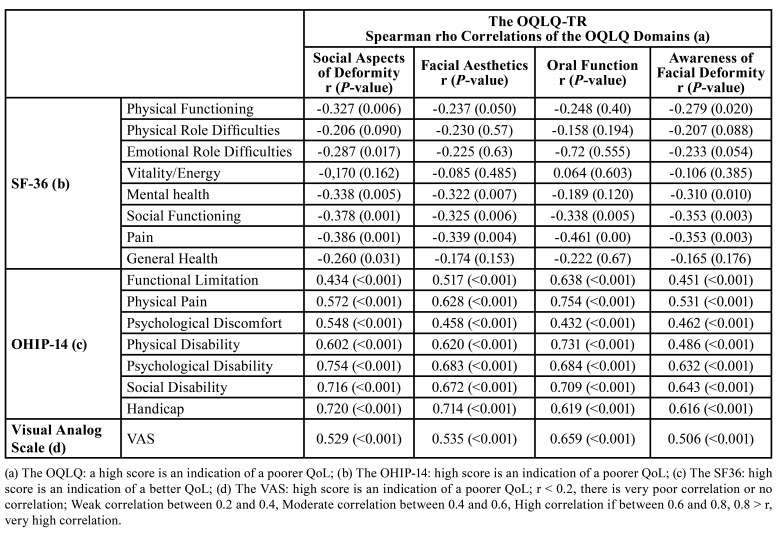
Relationships between the SF-36, OHI-14, and VAS of the OQLQ-TR.

## Discussion

One of the important health problems that affect the QoL of individuals in the field of dentistry are dentofacial problems. The main reasons why individuals want to be treated for their dentofacial problems are developmental and chronic conditions that result in social handicaps, such as abnormal facial appearances. Functional difficulties in chewing food, discomfort, and pain (particularly due to TMJ dysfunction) are also effective in the emergence of the need for treatment, but are often overshadowed by the social effects of the patient's facial appearance. Therefore, this is directly related to the issue of QoL (8,9,21-24).

In order to determine the QoL in patients who have DDF, the OQLQ scale was developed and applied by Cunningham *et al*. (2000, 2002) (10,11). To date, a number of studies have been carried out on this questionnaire. The reliability and validity of the OQLQ were found to be meaningful and it was also found to be sensitive in the discrimination of DFDs in previous studies (9-11). Therefore, it has been translated into many languages since its first publication in 2000 (12,14-17,24-26).

The reliability of the OQLQ-TR was excellent with a high Cronbach alpha value range between 0.873 and 0.944. While the Cronbach's alpha value for the overall score exceeded 0.9, the intraclass correlation coefficient was greater than 0.8 for all scores. In this study, ICC by dimensions was similar to the value reported by Cunningham *et al* (11). The Spanish version (13), the Brazilian version (12), the Serbian version (14), and the Chinese version (26), including the original scale (11), have Cronbach's alpha values for all four domains, similar to the results of the OQLQ-TR. The ICCs of the 4 domains of the questionnaire were evaluated based on the homogeneity of the responses to each item. A very high ICC was observed especially in the social aspects of the deformity domain and the consistency between the 1st and 2nd applications of the questionnaire was found to be high in all of the domains (Table 2). This finding meant that the survey exhibited reliability for repeated evaluations and high stability over time.

Various approaches have been applied to investigate the QoL of individuals who have DDF; however, there is no consensus with regard to a standard method of assessment, and the responsiveness of the generic measures that are used in the evaluation of oral conditions or diseases is limited, emphasizing the how important it is to improve oral condition-specific QoL measures (2,15).

Generic QoL scales often address areas of general well-being without focusing on specific diseases on their own, and their main advantage is their availability to compare various conditions that affect health-related QoL. Al Ahmad *et al*., Bortoluzzi *et al*., Cunningham *et al*., and Lee *et al*. observed that poor correlations existed between the domains of the OQLQ and the scales of the SF-36, similar to the present study (2,10,12,15). According to these findings SF-36 was insufficient in determining the difference in QoL between patients with and without DDF. It is believed that the use of condition-specific scales to score the QoL of patients with DDF is important for discrimination.

The OQLQ-TR was compared with the OHIP-14 to determine its validity. The correlations between all domains of the OQLQ-TR and OHIP-14 were all significant (Table 4). These findings were similar in all other studies using OHIP-14 for validity. and supported the fact that condition-specific QoL scales are highly compatible and the validity of the OQLQ-TR (2,6,12,25).

In line with the answers given by the patients on the VAS, the oral function domain of the OQLQ-TR produced the greatest correlation (0.659). Thus, the VAS scores supported the validity of the OQLQ-TR, but it was not sufficient alone, because the measure of a single item is not as reliable and is not able to capture the concerns of all patients.

All of the studies using the OQLQ which was developed by Cunningham *et al* (10,11), including the original version of the OQLQ, evaluated patients who started orthodontic treatment prior to orthognathic surgery. Since the present study is a methodological reliability and validity study, patients with DFD who had not been receiving orthodontic treatment were included in this study. However, the present study claims that starting orthodontic treatment is one of the significant factors that may affect the QoL of patients.

The OQLQ-TR has been demonstrated to be a simple and easy-to-use language questionnaire that provides the ability to be understood by individuals of any socio-cultural level. This also proved that the OQLQ-TR is reproducible, reliable, valid, and equivalent to the original source (the OQLQ). Thus, an important scale was gained for Turkish-speaking patients to assess the effect of DDF on the QoL of Turkish people.

## Conclusions

This study evaluated the QoL, and functional and psychological conditions of patients who had DFD, as well as the cross-cultural adaptation, validity, and reliability of the OQLQ-TR. The QoL of patients who have DFD can be clinically evaluated in Turkey by using the OQLQ-TR, whose validity and reliability have been proven by the present study, and various studies can be conducted on this subject.
